# Global reactivity models are impactful in industrial synthesis applications

**DOI:** 10.1186/s13321-023-00685-0

**Published:** 2023-02-11

**Authors:** Paulo Neves, Kelly McClure, Jonas Verhoeven, Natalia Dyubankova, Ramil Nugmanov, Andrey Gedich, Sairam Menon, Zhicai Shi, Jörg K. Wegner

**Affiliations:** 1 In-Silico Discovery and External Innovation (ISDEI), Janssen Research & Development, Janssen Pharmaceutica N.V, Beerse, Belgium; 2 Discovery Chemistry LJ, Janssen Research & Development, Janssen Pharmaceutica N.V, Philadelphia, United States of America; 3 Software Country, Tbilisi, Georgia; 4 Pharma R&D Information Tech, Janssen Research & Development, Janssen Pharmaceutica N.V, Beerse, Belgium

## Abstract

Artificial Intelligence is revolutionizing many aspects of the pharmaceutical industry. Deep learning models are now routinely applied to guide drug discovery projects leading to faster and improved findings, but there are still many tasks with enormous unrealized potential. One such task is the reaction yield prediction. Every year more than one fifth of all synthesis attempts result in product yields which are either zero or too low. This equates to chemical and human resources being spent on activities which ultimately do not progress the programs, leading to a triple loss when accounting for the cost of opportunity in time wasted. In this work we pre-train a BERT model on more than 16 million reactions from 4 different data sources, and fine tune it to achieve an uncertainty calibrated global yield prediction model. This model is an improvement upon state of the art not just from the increase in pre-train data but also by introducing a new embedding layer which solves a few limitations of SMILES and enables integration of additional information such as equivalents and molecule role into the reaction encoding, the model is called BERT Enriched Embedding (BEE). The model is benchmarked on an open-source dataset against a state-of-the-art synthesis focused BERT showing a near 20-point improvement in r2 score. The model is fine-tuned and tested on an internal company data benchmark, and a prospective study shows that the application of the model can reduce the total number of negative reactions (yield under 5%) ran in Janssen by at least 34%. Lastly, we corroborate the previous results through experimental validation, by directly deploying the model in an on-going drug discovery project and showing that it can also be used successfully as a reagent recommender due to its fast inference speed and reliable confidence estimation, a critical feature for industry application.

**Supplementary Information:**

The online version contains supplementary material available at 10.1186/s13321-023-00685-0.

## Introduction

A key aspect of chemical synthesis is to find efficient ways to prepare “sets of compounds” or libraries and to explore if they contribute to the design of new lead candidates. There is a clear role that data analytics and machine learning (ML) techniques have in the design/make/test cycle of medicinal chemistry, and that is also the case for synthesis specific challenges in the cycle, such as synthesizability estimation and condition recommendation amongst others, as discussed in [1]. In the last three years there has been tremendous progress in backward synthesis (given product → predict suitable reactants), forward synthesis (given reactants → estimate product), as well as retrosynthesis (multi-step backward) and library generation (large-scale virtual screening on single-step forward or multi-step forward), e.g. by doubling the accuracy on backward synthesis estimates. Almost all of those methods focus directly or indirectly in the estimation of regioselectivity, but do not tackle the core question “can this compound be synthesized in an isolatable yield?” or practical drug discovery questions like “which conditions can synthesize an entire exploratory 24 compound set?”. Estimating regioselectivity alone is insufficient to predict product yield and thus optimize success chances for a single or parallel synthesis experiment. Current approaches often rely on empirical “robust reactions” expert system rules e.g. DOGS [2], SAVI [3] and Enamine REAL. 1 Such expert-systems have an average success rate of around 80% and raise two main concerns. First, there is no relation between reactant or reagent combinations that would indicate a higher or lower chance of success. Secondly, there is no growth opportunity using industry-scale ELN data to improve business processes over time.

A review from Saebi et al. [4] highlights newer data analytics formulations using deep learning, such as yield estimates from Schwaller et al. [5] and Saebi et al. [6]. Our work addresses both core challenges showcasing that we can improve “reaction success” estimates based on an industry-scale ELN data integration retrospectively as well as prospectively. In contrast to historic “robust reaction” estimates with a static success rate, our formulation allows to estimate which reactant and reagent combinations will lead to higher success, which coupled with confidence estimates and fast inference speeds enables a reagent recommender formulation that can be used to optimize single or multiple reactions simultaneously in parallel medicinal chemistry exercises. The approach used in our work also enables future growth opportunities as the chosen data analytics formulation benefits from additional synthesis and condition data that becomes available over time. Although not used in our current approach, future data growth is not limited to experimental data alone, quantum chemistry simulation data can additionally improve scientific performance as shown by Guan et al. [7]. This enrichment is especially pronounced in regions of low data availability, improving few-shot learning capacity of reaction success estimations.

## State of the art

The percentage of isolated reaction yield is dependent on a plethora of factors beyond the representation of the reaction as reactants, reagents and products, and has a high dependency on the process conditions, which includes reaction parameters such as molar scale, temperature and concentration, as well as purification method and technology used. This high dimensionality points to the complexity of accurately modelling a “global” reaction space to predict sensible reaction yields, as evidenced by initial studies that used chemical descriptors and traditional ML methods [8]. A work whose resulting performance was not sufficient to fulfil the ultimate goal of enabling chemists to predict optimal conditions and prioritize compounds by highest chance of synthesis success. The modelling exercise is even more challenged by dataset bias [9] and inconsistently reported, heterogeneous data sources [10], containing both unoptimized transformations, e.g. with the purpose of isolating material for evaluation in a biochemical assay, and optimized protocols, e.g. in the development of new synthetic methodology or for scale-up purposes. In addition to that, reported examples of unsuccessful reactions, resulting in a 0% yield or traces of the desired product, are clearly underrepresented, yet equally important to enable the model to identify negative reactions.

These limiting factors hinder the use of diverse chemical reaction datasets, whose diverse conditions are not recorded in a consistent structured format, from being used to develop high-performance global yield prediction models. This conjecture in turn triggered investigations towards methodologies that allow accurate predictions on a more focused chemical space, e.g. covering transformations that are relevant in the pharmaceutical industry for which dense High-Throughput Experimentation (HTE) datasets exists. In this regard, the Doyle group investigated the predictive performance of random forest ML models trained on Pd-catalyzed Buchwald-Hartwig amination reactions using a dedicated HTE dataset. This study resulted in an improved predictivity model over linear regression analysis which has the potential to predict optimal reaction conditions for a new substrate undergoing such a transformation [11, 12]. The same dataset was used in [13], where a model using multiple fingerprints features obtained generally comparable results. To support the selection of reaction conditions that optimize the yield of reaction processes, the Doyle group developed a Bayesian optimization framework for integration in the laboratory. This approach outperformed human decision making in regards to efficiency and consistency when selecting subsequent improved reaction conditions for a palladium-catalyzed cross coupling benchmark [14]. Furthermore, Gao and co-workers developed a neural network model trained on a wide range of organic reactions (approximately 10 million records) from the Reaxys database, in an attempt to accurately predict chemical reaction context [15]. Here, a top-10 accuracy of 69.6% for predicted conditions that closely matched the ground truth on selected catalyst, reagent or solvent was recorded and the utility of the model was further demonstrated on a selection of commonly used reaction classes.

Assuming reaction condition data can be accurately captured from existing datasets or that new condition-structured datasets will emerge, the pursuit of a high-performance global yield prediction model is not unreasonable, and the development of novel artificial intelligence architectures and techniques have accelerated the creation of such a model.

In 2017, Vaswani et al. initiated a new wave of models based on the transformer architecture [16], that became the state of the art in natural language tasks. By stacking the encoder blocks from the transformer and using other techniques such as autoregressive pretraining protocols, a year later, Devlin et al. published Bidirectional Encoder Representations from Transformers (BERT) [17]. This work changed the paradigm of training to unsupervised pretraining of the model in very large datasets, which then could be fine-tuned with smaller datasets for specific downstream tasks. This approach has led to state-of-the-art results not just in the natural language processing field but also in many other fields such as image prediction tasks. By using the SMILES representation, chemical reactions can be represented in text format, which effectively enables the use of natural language processing techniques and models for the reaction space. In 2021, Schwaller et al. published an application of BERT with the regression downstream task for yield prediction [18]. This publication showed the great potential of transformer-based architectures in yield prediction by achieving an average R^2^ of 0.951 for a Buchwald–Hartwig high-throughput experiment dataset, performance which can be further improved by using data augmentation strategies [19].

The use of BERT for reaction fingerprint generation was further validated through a thorough benchmark study which demonstrated that the use of reaction SMILES alone as input for the BERT-based reaction encoder outperformed previous studies using DFT based chemical descriptors [11] or multiple fingerprint features (MMF) [13] in random-forest ML models for yield prediction tasks. Interestingly, a recent study by Probst et al. demonstrated that the differential reaction fingerprint (drfp), computed from circular substructures in the reaction SMILES [20], shows a competitive performance on the aforementioned benchmark dataset when compared to the deep learning extracted fingerprints.

Despite the encouraging results from the BERT-yield model, the performance on the USPTO dataset for a time split train-test was not sufficient for application in industry use cases. The authors point out that the poor performance might be related to data quality issues in regard to the yield values. Although this is a valid point that should be investigated, we believe that it is not possible to construct a high-performance global yield prediction regression model from the reaction SMILES only. As pointed out previously, and supported by discussions with chemists at Janssen, the importance of conditions and process information e.g. temperature, equivalents, concentration , reaction time, heavily impacts the reaction yield. Consequently, in a dataset such as USPTO, containing only positive reactions from multiple data sources, the model does not receive the information accounting for the protocol signal and is unable to accurately predict quantitative values regardless of structure or even mechanism of the encoding method. Due to this limitation, we attempt to tackle the global yield prediction challenge as a binary classification task where the goal is to predict if a reaction will result in a yield which is less or greater than 5%. Being able to accurately predict which reactions will succeed and as a result, limit the execution of negative reactions in the lab is a valuable asset for resource optimisation in Structure-Activity Relationship (SAR) exploration. Furthermore, being able to suggest a set of reagents which will improve chances of synthesis success when the model predicts failure might even unlock compounds which would otherwise not be synthesized. This binary classification task is tangled with a core objective in medicinal chemistry programs, to isolate the compound in a sufficient amount for biological testing which is often more important than optimising an isolated yield for an already successful reaction.

## Methods

We use the code and approach published in [18] as ground zero for our research. Knowing that it would not be possible to build a high-performance global yield regression model with available data we adapted it to a solve a binary classification task by replacing the regression layer with a classification layer at end and using cross-entropy loss. However, as it will be shown later, we discovered that a regression task between 0 and 1 enabled an inexpensive calculation of uncertainty which was better calibrated than the confidence score extracted from the SoftMax function of the classification model. As will be discussed later, this further enables other business use cases and translates to a higher performance in practice.

For the precursor model the work from [18] used the BERT model trained in [21], which had as its training dataset 2.6 M reactions extracted from the Pistachio database, going forward this model shall be referred to as the Pistachio pre-trained regression model. In our work we extracted 16 M reactions from Reactlake which is a Janssen database that combines multiple data sources as discussed in the following section. We also optimized some of the hyperparameters towards our pretrain and train dataset and then devised a new embedding layer to make use of available information such as molecule role, equivalent data and to solve some limitations of using SMILES with BERT models. The model containing this layer will be referred to as Bert-Enriched-Embedding (BEE).

### Data sources and data standardization protocol

For this work we used Janssen’s internal Reactlake reaction database. This database is composed of the open source reaction datasets USPTO [22] and commercial reaction datasets, Reaxys www.reaxys.com and Pistachio https://www.nextmovesoftware.com/Pistachio.html and Janssen’s internal proprietary data, Janssen ELN, which in this work is separated from Reactlake to be used in a different stage of training. The data in Reactlake is standardized in the same way as described in [23], this protocol involves aromaticity fixing, functional groups transformation into single representation form and atom-to-atom mapping. For atom-to-atom mapping RxnMapper[24] was used while the molecules processing was executed with the CGRtools package [25].

After combined and duplicated reaction smiles removed, USPTO, Reaxys and Pistachio had about 15.5 million unique reactions. Although the combined dataset was heavily skewed towards reactions above > 5% yield, this dataset was only used for masked language modelling (MLM) where the model learns the probability distribution of each token relative to available token context, as such, it is beneficial that 0% yield reactions are not provided to the model such that impossible or very unlikely token probability distributions are not learned.

As for the processed Janssen ELN used for fine-tuning, a sub-selection of 750 K unique reactions with a class distribution of about 24% negatives and 76% positives was used. Some noteworthy characteristics of this dataset are that each entity usually has a role associated with it and that entities are innately separated.

### Models and training protocol

The models are based on the models from [18]. The pretraining is executed using only MLM on Reactlake SMILES, excluding Janssen ELN. We increased the max sequence length to 550 elements such that the vast majority of reactions smiles in the training datasets aren’t capped during training. Batch size was also increased to make full use of the GPU memory capabilities and starting learning rate was adjusted linearly in accordance, both for pretraining and finetuning. Since Reactlake data does not consistently contain molecular ratio and concentrations we opted to set the additional embedding layer of BEE to zero during the MLM. While maintaining most hyperparameters constant we attempted increasing the number of attention heads to 32 and the size of the hidden layers to 512 but after convergence we verified no benefits for downstream tasks.

The finetuning also follows the protocol from [18], but regression is done between 0 and 1, in this step only Janssen ELN data is used for the yield prediction binary classification task. For each input reaction smiles a condition’s Id vector is generated with the same size, such that every token in the reaction smiles corresponds to an Id. Ids are categorical values that can be generated automatically or manually and depend on the equivalent/molecular ratios/concentration threshold or molecular roles the user defines. Grid search was used to optimize two hyperparameters, starting learning rate and dropout value used for the finetuning task.

### Uncertainty estimation and its importance in industry application and business cases

Uncertainty estimation is a crucial consideration for real world applications of deep learning. When selecting the best reagents or substrates to achieve a specific product, a chemist can use the model’s success estimation prediction and uncertainty calculation to probe the reaction space successfully covered by the model, to a certain degree, with different reagent and/or starting material combinations until finding a positive prediction with low uncertainty. This enables use cases of the model with significantly higher performance than what the global performance in the benchmark indicates.

**Fig. 1:**
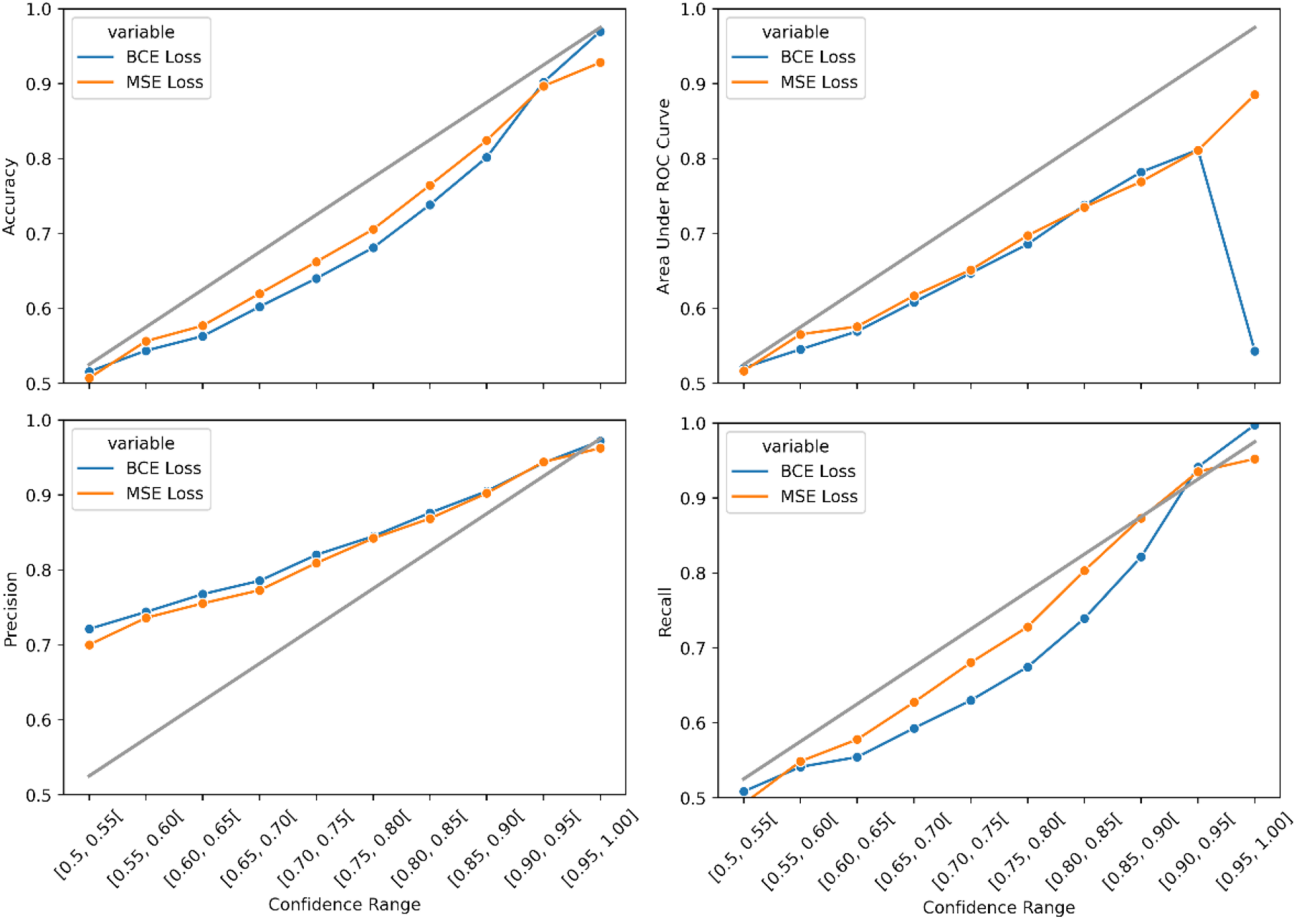
Network calibrations for different performance metrics on the 2021 internal chronological benchmark, for binary yield classification task

For this reason, we include a very inexpensive calculation of confidence defined by $2\Vert x-0.5\Vert$, where $x$ is the numeric prediction, the result is inversely correlated with uncertainty such that the higher the confidence the higher performance we can expect from the model. Given that the model performs a binary classification task where in a balanced class setting, the minimum expected accuracy is 50% and the maximum is 100%, in \* MERGEFORMAT Fig. 1 confidence is linearly scaled to values between 0.5 and 1. This way a perfectly calibrated confidence curve will have a slope of 1 and start at the point (0.525, 0.525) for each of the performance metrics.

The measure of confidence and performance displayed in \*MERGEFORMAT Fig. 1 derives from a chronological validation exercise where the downstream model was trained and validated with a subset of Janssen ELN data only generated up to 2020 and subsequently tested on 2021 Janssen ELN synthesis data. It’s worth noting that a high precision on the prediction of the positive class (yield >  = 5%) is to be expected as the test set is considerably skewed towards this class.

There are two lines presented in each graph of \* MERGEFORMAT Fig. 1. These refer to two Reactlake BERT models, one showing performance per each range of confidence values provided by the SoftMax function at the outer layer of the classification model which uses cross entropy loss. The other line labelled as MSE Loss is defined by the performance of the model which predicts values between 0 and 1, and uses the difference of the predicted value to the actual class to generate a confidence value between 0.5 and 1. In this task all reactions with yield below 5% are set to 0, while the rest are set to 1. The four performance metrics are calculated simultaneously for each batch of reactions corresponding to a specific confidence interval outputted by the model.

Even though the use of such confidence scores is known to be miscalibrated [26–28] as shown in [29] an application of transformers in synthesis modelling and corroborated in this work, it still provides large value for an out-of-the-box technique with zero additional cost. The same cannot be said of other superior confidence estimation performance methods such as Monte Carlo Drop Out, which require multiple forward passes at each prediction, or Deep Ensembles which also suffer from additional compute cost considerably slowing the speed performance of the models and thus removing applicability in certain business use cases. Inclusion of these methods can be considered for future work, where depending on the use case different uncertainty estimation techniques are employed according to time constrictions and available compute power.

The largest discrepancy between the methods occurs in the ROC AUC at the [0.95–1.00] confidence range where the AUC value is 0.5431, however this result derives from a miss labeling of less than 0.14% of the data and as such it is a negligible result. More concretely the percentage of the total test data in the [0.95–1.00] range for the Reactlake classification model is 4.73%, out of which the negative reaction data represents a mere 0.14%. Although metrics such as accuracy, precision and recall on the positive label are practically unaffected by incorrect labeling of the negative class when this one is so underrepresented, for a metric such as the ROC AUC which is a function of false positive rates, a large percentage error in a heavily unrepresented class still has a very large impact.

### Equivalents enriched embedding layer

There are many avenues that can be pursued to improve deep learning reactivity models, from integration of quantum mechanics descriptors, new methodologies for reaction representation, introducing new pretraining protocols or model architectures to name a few. However, chemists primarily recognize the paramount importance of reaction conditions when optimizing reaction yield. Particularly in high throughput experimentation, an array of examples can be found which show for the same chemical reaction the yield varying from 0 to 85% by only selecting different equivalents of the building blocks or additives, e.g. by modulating the excess of base and reagent [30], or by selecting different temperatures and reactions time, which optimized reaction yield from 40 to 100% in [31]. For this reason, we prioritized the integration of equivalent information into the model through a new embedding layer that aims to address other limitations of current synthesis models using SMILES.

1. The most important contribution of our approach to improving reactivity prediction is the introduction of the molecular ratios into the new embedding layer. The knowledge of the molar ratio between reagents and the limiting reactant (entity which restricts the amount of yielded product by being consumed first) enables a more fundamental chemical differentiation with respect to learning the reactivity of a reaction.
2. One of the limitations introduced by the SMILES representation is that molecule separators “.” are also used to imply ionic bonds. With the new embedding layer, the molecule separation role of “.” becomes distinguishable from ionic bonds by receiving a different representation, which also results in a continuous representation of these types of molecules, e.g. salts. In our experiments, correct id attribution to differently purposed “.” did not require an algorithmic solution as Janssen ELN innately has molecular entities already separated.
3. In natural language text tasks such as question answering, “segment id” is used in the embedding of transformer-based models to help distinguishing the question from the answer, reactions can be viewed in a similar way where the left side is a question and the right side is the answer, however until now this distinction had not been implemented in the embedding layers of SMILES based synthesis transformers.
4. The equivalent class represented in the embedding can be used to convey the molecule role when equivalent information is missing, which is common for solvents, for example. In such a conjecture the model has the ability to identify the role of the molecule. This is relevant to the outcome of the reaction in multiple ways such as knowing that certain molecules will not contribute atoms to the product, or that changing a solvent or a catalyst can have a specific and different impact on reactivity in an otherwise similar reaction.

It is important to note that points (2), (3) and (4) referred above are only relevant when there is limited data, as otherwise a transformer model will generally be able to learn to distinguish tokens “contributions” from context.

**Fig. 2:**
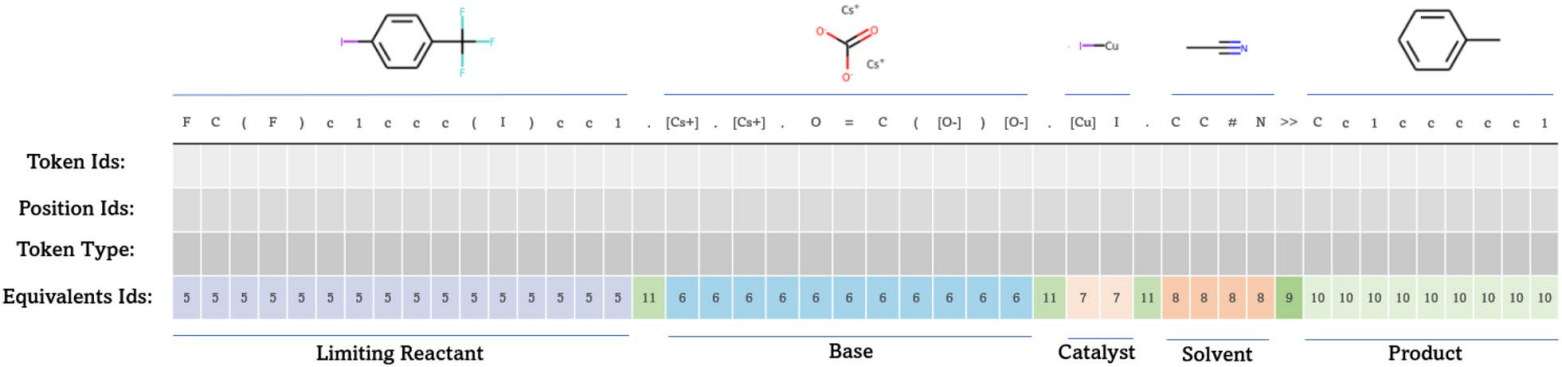
Random reaction showing the conversion of smiles to embedding ids in the new embedding layer

In Fig. 2 an example of the conversion of molecules to embedding ids is provided. This example highlights how the token “.” separating molecules (Id 11) is represented with a different id compared to the token “.” present in the base (Id 6). This means the entire base, including the ionic bond is represented with the same Equivalent Id creating a consistent contribution of the base to the reaction embedding. All tokens from the product and “ >  > ” will constantly be represented with Id 9 and 10 which makes the role of the product even more explicit. Solvents and limiting reactant also have a constant id, to support learning the type and impact of their role. Lastly any other reagents such as bases, catalysts and other additives will be defined by their equivalent class which can vary. This enables the model to learn how the same molecule can have different impact on the reactivity as a function of the molar ratio it has relative to the limiting reactant.

As indicated by Fig. 2 Equivalent Ids are categorical values, much like molecule roles, while molar ratio, moles and concentration will be numerical values. Different datasets can have access to a wide range of condition information, so chemistry knowledge can be used to define thresholds for the numerical variables such that these can be converted to classes (Ids). Distributions of these numerical variables can also be used to determine the appropriate thresholds.

Since the data used for the pretraining did not contain this type of additional condition information, the new embedding layer is set to null during this training stage, however the size of the embedding layer already needs to be defined such that when finetuning is initiated a placeholder in the network for the additional information already exists.

During finetuning the embedding becomes enriched by doing the sum of the vectors learned for each “Equivalent Id” with the normal embedding, this sum can add information about, molecule role, molar ratio, moles, concentration, token differentiation, left and right side differentiation or any other condition/procedure data that can be converted into a categorical variable and provided for a specific entity. In fact, full reaction data, such as temperature or time can also be provided to the model with this approach either through the categorical “Equivalent Ids” or through another additional embedding layer.

## Results and Discussion

**Fig. 3:**
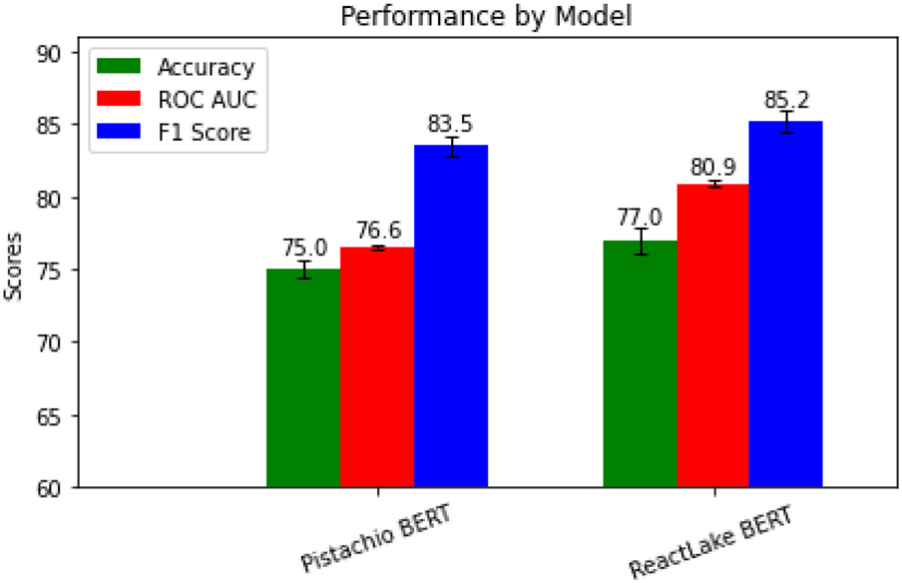
Bar plot showing the performance on Accuracy, ROC AUC and F1 Score for models pretrained with MLM and then finetuned on Janssen ELN to solve a reaction global binary classification task of yield prediction. Models presented are Bert pretrained on Pistachio, compared with the same model but pretrained on Reactlake using the code for BEE with the enriched embedding disabled

Two models have been trained and compared (Fig. 3). The first, denoted as Pistachio Bert is the pretrained model published in [18]. This is a state-of-the-art global yield prediction model which we fined tuned and tested using Janssen data. The second model denoted as a Reactlake Bert (Fig. 3), is a BERT model pretrained from scratch using the Reactlake data and undergoing the same finetuning train and test protocol as the previous model. From this result we observed that pretraining BERT on a significantly larger dataset can have a positive impact in performance of the downstream task.

To measure performance, we applied Nested Cross Validation splitting Janssen internal dataset into 7 slices and ran 7 iterations of train optimization-validation and test. In each iteration five slices of the data are used for training, while a 6th slice is used to perform early stopping and hence select the best epoch. The model trained on that respective epoch is then tested on the 7th slice which was hidden during training, validation and epoch selection.

It is important to note that for previous benchmark we did not use the enriched embedding as in the context of historical medicinal chemistry data, reactions don’t often have yield under 5% due to incorrect choice of equivalents. Additionally due to high variations of yield for reactions with the same molecules, variations which are caused by many external factors, extracting the signal of the equivalents in a global dataset is challenging. To make evident the advantages of using an enriched embedding with some condition information we used a published dataset [32] designed and executed specifically to provide a high-quality, consistent and relevant data to train and test machine learning approaches.

**Fig. 4:**
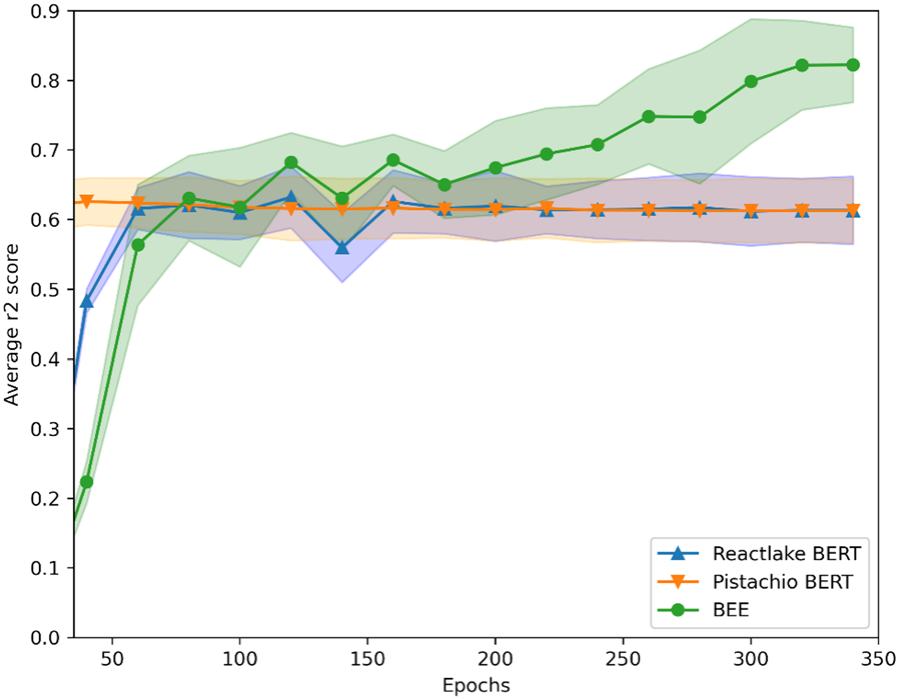
Scatter plot showing the average r2 score across the test splits of a 5-split nested cross validation protocol, on an opensource high-quality dataset with 1728 C-N coupling Photo-Redox reactions

\*MERGEFORMAT Fig. 4 shows how the Pistachio BERT, Reactlake BERT and BEE performed when fine-tuned for a regression prediction task on the dataset, interestingly for the specific reaction type in the dataset the larger pretrain dataset of Reactlake did not provide an advantage over the use of Pistachio, however there is a large difference of almost 0.2 in r2 score between BEE average performance and the average of other methods, indicating the importance of learning and including impactful reaction condition information like equivalents.

An aspect which is sometimes neglected in AI publications of novel models and algorithms is the speed performance of the model. In an industrial context many business use cases can require multiple millions of predictions to be executed in a tractable amount of time, as such the speed performance of a model can be the difference between having real-world applicability or not. In a machine with a V100 GPU with 32 GB of memory, BEE is capable of performing an average of 530 inference operations per second. This enables the model to be applicable in use cases that require up to 45 million predictions a day. Speed is a critical characteristic for business use cases, for example, to design a 24 well plate a chemist might consider 5000 building blocks for R-Group substitution on the project’s main scaffold and for some reactions we can have 1000 different conditions. With a slow technique testing each condition in-silico for a given building block-scaffold pair would be impossible as 5 M predictions would take too long, but with this approach it will take under 3 h. This is essentially a paradigm shift since it means we can consider synthesizability as a property when doing multi property optimization based design of the plate, while selecting a set of reagents which optimize the entire 24 well plate instead of a single proxy reaction, as it is commonly done in the present.

### Model validation 2021 chronological test split

In addition to the Nested Cross Validation, we evaluated the impact BEE would have had at Janssen in 2021 if it had been available and used diligently by the chemists, as an interesting business performance metric.

In this prospective exercise we trained the downstream model using internal Janssen ELN data collected from 2007 to 2020 and reserved all data from 2021 as the test set. This allows to accurately calculate how many reactions that resulted in yields below 5% would have been flagged by the model, thus allowing the chemists to pre-emptively review other synthesis options. Furthermore, for the synthesis review the chemists could then use the model to run in-silico experiments in order to find the reagent conditions predicted to have the highest ch**a**nces of avoiding a failed reaction.

**Fig. 5:**
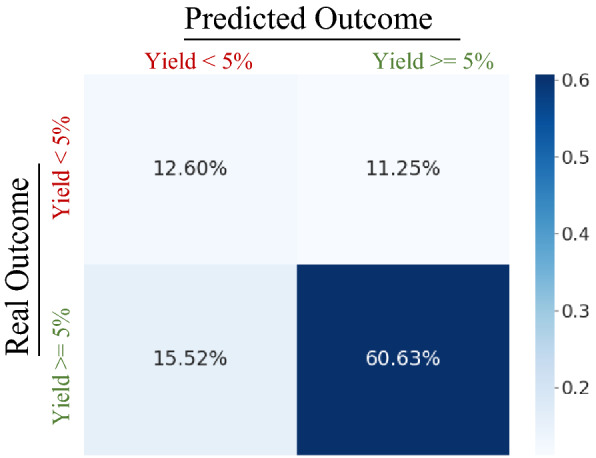
Normalized confusion matrix for predictions of BEE in the 2021 test set while fine-tuned for yield classification Janssen ELN synthesis data up to 2020

Figure 5 contains the resulting confusion matrix showing the performance of the model for both classes in the 2021 test. Using the normalized confusion matrix, we observe that out of all the reactions in the 2021 test set 23.85% were negative reactions. The following formula allows us to calculate an estimate of the percentage reduction in negative reactions that would have been prevented in 2021:

$$1 - \frac{{1 - T.P\sum\nolimits_{i = 0}^{n} {(T.N + F.N)^{i} } }}{T.N + F.P}$$

T.N – True Negatives;

F.P – False Positives;

F.N – False Negatives

T.P – True Positives;

The *n* in the summation represents the number of additional design/test in-silico iterations the chemist would do before executing a reaction predicted to be negative. The formula takes into account that despite the model’s ability to immediately flag correctly 52.8% of all negative reactions, all negative predictions should be replaced with new reaction suggestions, at which point the subsequent prediction of reaction success has again a probability of being negative, this probability is assumed as T.N + F.N. This leads into a loop where negative predictions require further alternatives-predictions that would generate other negative predictions.

For the purposes of estimating impact, we consider *n* = *4*. Which using the formula Additional File 1 indicates that 34.5% of all negative reactions ran by Janssen and partners in the year 2021 could have been prevented. This result has massive resource implications to any field running challenging and numerous chemical reactions much like the pharmaceutical industry.

Furthermore, thanks to the reliable confidence estimation and fast inference speeds of the model already discussed, the process of considering different reagents can be automated by a python script which given a set of reagents enumerates all possible combinations, creating an in-silico HTE plate which can have dozens of thousands of wells for the model to test and rank.

Although the time and financial cost of a given reaction can highly vary, it is common in drug discovery projects to explore multiple conditions before a suitable set can be found, a process which can take from one to 4 weeks, or even longer if the target is deemed valuable enough to commit to a longer timeframe of discovery. This results in reduced compound output from the chemists in the project, slowing down the progress of a program. The more costly negative reactions occur in cases where preparation of target molecules takes multiple steps, if failed reactions occur towards the end of the synthetic route, it can be exceptionally problematic. Thus, having a reliable model capable of quickly verifying each reaction step and conditions in advance for a given set of substrates becomes invaluable for medicinal chemists.

### Experimental validation

The chronological validation is a prospective exercise which accurately represents how the model would have performed in 2021, however the deployment and actual use of the model in on-going projects not only allows the corroboration of performance it can also demonstrate its usability as a reaction failure detector and as a reagent recommender.

In the project where the model was applied, chemists were having difficulties optimizing a copper catalyzed C-N coupling reaction, specifically, an HTE plate was executed where around 80% of the reactions had very low yield (< 5%). To use the model as a reagent recommender, the chemist defined a file with starting material and desired product, as well as another file containing 12 ligands, 3 solvents, 5 bases and 1 catalyst. A python script enumerating all 180 possible reagent combinations was subsequently combined with the starting material to the desired product to generate all possible reactions. The reactions were standardized using the same method applied to the train dataset and were then used as input for model inference. This corresponds to 180 different virtual attempts for each of the 40 unique combinations of starting material to product, giving a total of 7200 virtual reactions to be virtually screened by the model. The positive predicted reactions were separated from the negative predicted reactions and the confidence score was used to rank order. Given the correlation between historical data and a high confidence score in reactions equal or similar to the ones used in training, the model can be interpreted, in some ways, as the collective experience of the chemists who contributed to the internal Janssen ELN database. In other words, if one chemist learns that a specific reaction doesn’t work, all other chemists who use the model become automatically aware of this in the future.

Out of 25 reactions predicted to succeed with high confidence (> 0.75) by the model, only 2 resulted in yields below 5%, leading to a 92% success rate, this was a major improvement when comparing to the original HTE plate with 20% success rate. As a result, the chemistry team in the project was able to synthesize a lot of the desired compounds it needed without need for further HTE experiments.

**Fig. 6:**
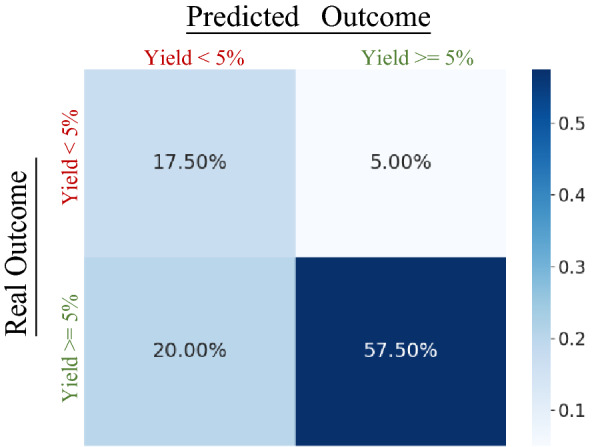
Normalized confusion matrix for Reactlake BERT executed predictions on reactions which were later executed experimentally

Having achieved the desired set of compounds, the team committed an extra effort to run reactions predicted by the model to fail, with the aim of having a negative control for validation of the model. As shown in Fig. 6 and Table 1 the performance of the model in the experimental validation was comparable or superior to the one estimated with the chronological validation for the Reactlake Bert which had been used to run the experiment as the effort to use enriched the embeddings was still on going. When comparing results with the performance in the buckets between [0.75–0.8[ and [0.8–0.85[ in \* MERGEFORMAT Fig. 1, we observe that recall was approximately 2% under, while precision and ROC AUC were approximately 6% and 4% higher respectively.

**Table 1:** Model performance in experimental validation when using predictions with an average of 0.8 ± 0.06 confidence

	ROC AUC	Accuracy (Label 1)	Recall (Label 1)	Precision (Label 1)
Experimental validation Mean Confidence 0.801 Standard Deviation 0.064	0.760	75.0%	74.2%	92.0%

## Conclusion

In this work we detail a method for global yield prediction, capitalizing on industry-scale ELN data, which can be used to significantly raise discovery productivity in industries synthesizing novel compounds, by avoiding failed reactions and recommending reagents for single and parallel synthesis experiments. This method was validated using prospective, experimental, and nested cross validations, including a benchmark of BEE on a regression task using an open-source high-quality dataset, where it greatly outperforms a state-of-the-art technique, corroborating our argument that inclusion of impactful condition information is critical. This formulation also enables future growth opportunities as it benefits from any additional synthesis and condition data that becomes available over time, as the produced data becomes more refined and structured.

### Study limitations and future work

In this study we present a binary classification global model with a useful confidence score, as well as a high-performance regression local model. The detailed approach enables chemists to avoid very low yield reactions and can act as a reagent recommender for single and parallel experiments through reagent combination ranking. However, to realize its full applicability potential, the model will have to be able to perform global regression tasks or at least a multi class classification task, e.g. being able to differentiate between 5%, 25%, 55%, 85% yield, such that it can be used for yield optimization use cases via the formulation of different reactant-reagent and conditions combinations. However, as indicated previously, the creation of a high performing global regression model requires greatly increased data quality and the inclusion of all useful procedure information signal. To achieve this, techniques which can parse procedure text into a structured format or encode it into the embedding will likely be significant. Additionally, techniques to identify and recommend which data production exercises are needed to bring the most performance with the least amount of data will also be important in the pursuit of a high-performance global regression/multi-classification model.

## Supplementary Information

**Additional file 1: **Supporting Material.

## Data Availability

Data and code which could be made available is available at https://github.com/PauloNeves-Git/BEE. Most of the work reported in this paper uses highly sensitive and proprietary data. For this reason, the data and consequently the models trained on that data cannot be made public. However, we believe in the importance of information dissemination and scientific reproducibility, as such, some of the crucial experiments shown on the paper were reproduced with open-source data. Both the notebooks with these experiments and the code necessary to train BEE are available on the aforementioned GitHub link.

## Footnotes

1 https://enamine.net/compound-collections/real-compounds/real-space-navigator
