# Correction: Global reactivity models are impactful in industrial synthesis applications

**DOI:** 10.1186/s13321-023-00705-z

**Published:** 2023-03-02

**Authors:** Paulo Neves, Kelly McClure, Jonas Verhoeven, Natalia Dyubankova, Ramil Nugmanov, Andrey Gedich, Sairam Menon, Zhicai Shi, Jörg K. Wegner

**Affiliations:** 1 In-Silico Discovery and External Innovation (ISDEI), Janssen Research & Development, Janssen Pharmaceutica N.V, Beerse, Belgium; 2 Discovery Chemistry LJ, Janssen Research & Development, Janssen Pharmaceutica N.V, Philadelphia, USA; 3 Software Country, Tbilisi, Georgia; 4 Pharma R&D Information Tech, Janssen Research & Development, Janssen Pharmaceutica N.V, Beerse, Belgium

**Correction** **:** **Journal of Cheminformatics (2023) 15:20** **https://doi.org/10.1186/s13321-023-00685-0**

Following publication of the original article [1], it was noted that due to a typesetting error, a few typos were introduced.

The original article has been corrected.
