# Supplementary material for: Global reactivity models are impactful in industrial synthesis applications
Source: J Cheminform. 2023 Feb 11;15:20. doi: 10.1186/s13321-023-00685-0 (PMC9921076; doi:10.1186/s13321-023-00685-0)
Supplement: Supplementary file 1 — Additional file 1: Supporting Material. [file 13321_2023_685_MOESM1_ESM.docx]

## Additional file 1 Material

Since the work reported in this paper uses highly sensitive and proprietary data, the models and data could not be made available. However, an open code repository was uploaded to Github, which allows the reproduction of the most important results using USPTO, an open-source dataset, for the pretraining of BEE and BERT and a high-quality HTE dataset for the finetuning yield-proxy prediction task. Figure S1 shows the result of comparing the two methods with open-source data.


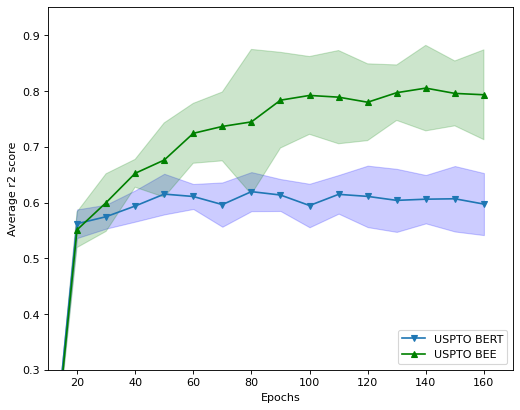


Figure S1- BEE and BERT are compared using the same pretraining dataset and a high-quality dataset for finetuning which has equivalent data available.

To estimate the percentage of negative reactions which would have been avoided in 2021 had the model been used diligently we make one assumption which allows infer the following formula.


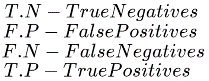

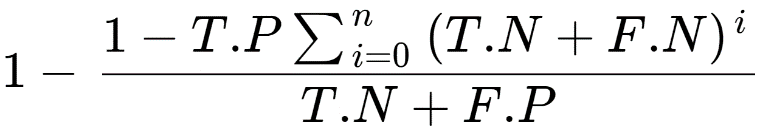


Figure *5* shows how the model would have performed in 2021 if it had only access to data from 2020 and years prior to train, without retraining throughout the year or using the confidence estimation to improve effective performance. In a hypothetical scenario where every chemist would use the model to do an in-silico test run before executing the reaction in the lab, 4 outcomes would occur. Reactions with T.P predictions would be successfully executed upon model confirmation, and F.P (both chemist and model assume reaction will work but it fails) would also be executed leading to failed synthesis. The rest, T.N+F.N would require the chemist to come up with an alternative reaction (eg, by changing conditions).

| Equation | Explanation |
| --- | --- |
| 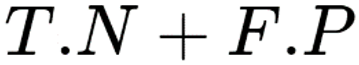 | Total percentage of confirmed failed reactions in 2021. |
| 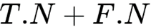 | Total percentage of negative predictions in 2021. All negative predictions would trigger a request for alternatives. |
| 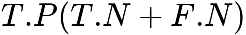 | Percentage of alternatives which would be correctly predicted to succeed. |
| 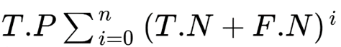 | Total percentage of hypothetical successful reactions in 2021, assuming that chemists would find a reasonable alternative for every negative prediction, and that the confusion matrix for the alternative reactions would stay constant. “n” being the number of times a chemist would try to find an alternative before running a reaction predicted to fail. The search for an alternative can be as simple as editing the reagents in the input and re-running the model. |
| 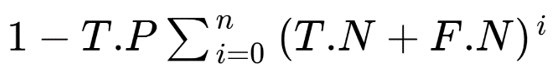 | Total percentage of hypothetical failed reactions in 2021, under assumptions defined above. |
| 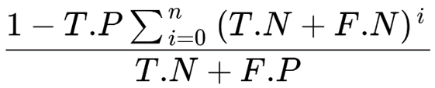 | Percentage of total negatives which hypothetically will still fail. |
| 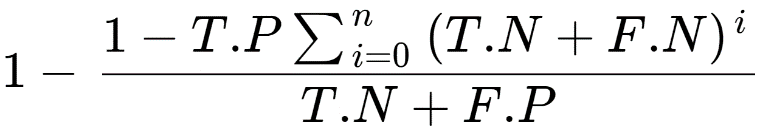 | Percentage of total negatives which would hypothetically be prevented. |
